# Public Health in the United States.—I

**Published:** 1906-01-06

**Authors:** 


### PUBLIC HEALTH IN THE UNITED STATES.—I.

Public health in the United States, as in all other countries, has been a question of evolution from small beginnings, and is of comparatively recent growth. The laws relating to this subject enacted by the general government and by the States reflect the attitude of the people. The number of such laws which appear in the early history of the colonies is not large. It is a matter of interest, however, to know that the early settlers soon after arriving in America recognised the need of definite knowledge on this subject, and of preserving their records, which constitute the foundation-stone of public hygiene, by enacting a law in 1639, that "there be records . . . of the days of every marriage, birth and death of every person in this jurisdiction." Progress in this direction during the first two centuries of the new nation's growth was slow and proportionate to the sparsity of the new settlements, density of population, from necessity constituting a prime factor in promoting public health measures. Up to the close of the eighteenth century, and for several decades of the nineteenth, almost the only health legislation which was enacted in the different States of the Union consisted in a few laws relating to small-pox, since this pestilence was scarcely ever absent for many years at a time from any city, town, or village, till after the general introduction of vaccination.

A commission was appointed on July 3rd, 1849, by the Governor of Massachusetts to make a sanitary survey of the State and to report upon the same. The report of that commission was an extremely valuable document, and though several years elapsed after its publication before a general board of health was established in any State, yet when such boards were established, the general plan laid down in this report was adopted, and now appears essentially in the organic acts establishing such State boards throughout the Union. The first three State boards of health were organised in three widely separated States: Louisiana, Massachusetts, and California, in the order named; and these were followed by the establishment of similar general boards in Virginia, Minnesota and Michigan.

As social civilisation has proceeded from the less to the greater, from the town and city to the country, and from the aggregation of these to the States, and then to the general Government, so also the history of sanitary organisation shows a similar order, the town board being the earliest unit of sanitary authority, then the State boards, and finally the national board which was not organised till 1879. The city or town board of health is and always has been the most firmly established organisation, and is usually clothed with the most arbitrary powers for the protection of each local community or municipality. In general it may be said that the work of State boards of health has not been largely of an executive character, but has been eminently didactic, and much good has been accomplished by the publication and distribution of tracts, circulars, and pamphlets relating to the various departments of public health, and by the holding of frequent conventions or assemblies for the free discussion of sanitary subjects. As a general rule, State boards of health do not have authority over local boards in sanitary matters, but in some instances are authorised to exercise co-ordinate power with them in preventing the spread of infectious diseases either within the limits of municipalities or along the border of other States or countries. The most important branch of public hygiene is the management and control of infectious diseases, and while the State boards of health are, from their necessary composition, not so closely in touch with the people as the municipal boards, yet they are capable of doing excellent service in educating the people in this important sanitary question. In those States which are the most densely populated, and are of comparatively small area, it has been possible for the general boards to perform a considerable amount of executive work, and to carry out the provisions of such laws as have given them authority to act for the protection of the public health. In several of the States, notably those of the western part of the Union, the function of regulating the practice of medicine has been added to the more distinctive duties of public sanitation.

At the present time the United States is without any general health organisation representing the national government which can in any manner be compared with those of the European countries—for example, the Comité Consultatif d'Hygiène of France, the Imperial Board of Health of Germany, or the Local Government Board of England.

(*To be continued.*)

